# A feasibility study of the use of UmbiFlow™ to assess the impact of heat stress on fetoplacental blood flow in field studies

**DOI:** 10.1002/ijgo.14480

**Published:** 2022-10-10

**Authors:** Ana Bonell, Valerie Vannevel, Bakary Sonko, Nuredin Mohammed, Ana M. Vicedo‐Cabrera, Andy Haines, Neil S. Maxwell, Jane Hirst, Andrew M. Prentice

**Affiliations:** ^1^ Medical Research Council Unit The Gambia at London School of Hygiene and Tropical Medicine Banjul The Gambia; ^2^ Centre on Climate Change and Planetary Health London School of Hygiene and Tropical Medicine London UK; ^3^ Maternal and Infant Healthcare Strategies Unit, SAMRC Pretoria South Africa; ^4^ Department of Obstetrics and Gynaecology University of Pretoria Pretoria South Africa; ^5^ Research Centre for Maternal, Fetal, Newborn & Child Healthcare strategies University of Pretoria Pretoria South Africa; ^6^ Institute of Social and Preventive Medicine, University of Bern Bern Switzerland; ^7^ Oeschger Center for Climate Change Research University of Bern Bern Switzerland; ^8^ Department of Public Health, Environment and Society, Department of Population Health London School of Hygiene and Tropical Medicine London UK; ^9^ Environmental Extremes Laboratory University of Brighton Eastbourne UK; ^10^ Nuffield Department of Women's and Reproductive Health and the George Institute for Global Health University of Oxford Oxford UK

**Keywords:** Africa, climate change, fetoplacental circulation, heat, pregnancy

## Abstract

**Objective:**

To evaluate the use of UmbiFlow™ in field settings to assess the impact of heat stress on umbilical artery resistance index (RI).

**Methods:**

This feasibility study was conducted in West Kiang, The Gambia, West Africa; a rural area with increasing exposure to extreme heat. We recruited women with singleton fetuses who performed manual tasks (such as farming) during pregnancy to an observational cohort study. The umbilical artery RI was measured at rest, and during and at the end of a typical working shift in women at 28 weeks or more of pregnancy. Adverse pregnancy outcomes (APO) were classified as stillbirth, preterm birth, low birth weight, or small for gestational age, and all other outcomes as normal.

**Results:**

A total of 40 participants were included; 23 normal births and 17 APO. Umbilical artery RI demonstrated a nonlinear relationship to heat stress, with indication of a potential threshold value for placental insufficiency at 32°C by universal thermal climate index and 30°C by wet bulb globe temperature.

**Conclusions:**

The Umbiflow device proved to be an effective field method for assessing placental function. Dynamic changes in RI may begin to explain the association between extreme heat and APO with an identified threshold of effect.

## Synopsis

Extreme heat exposure is increasing and a low‐cost umbilical artery Doppler device, UmbiFlow™, can aid understanding of fetoplacental function under heat stress conditions.

## 1 INTRODUCTION

With the ongoing climate crisis, global extreme heat exposure is progressively increasing with, for example, 30% of the world's population already exposed for 20 or more days annually to levels of heat sufficient to cause excess mortality and up to 74% predicted to be exposed by 2100.^1^ Sub‐Saharan Africa, South Asia, and Southeast Asia have been identified as regions at high risk of climate‐change‐related extreme weather events, despite contributing almost nothing to the problem.^2^ In The Gambia, West Africa, extreme heat, defined as above the 90% centile compared with the average temperature for that region (>39.4°C), occurred on average for 50 days per year, from 2016 to 2019 (from local weather station data). The double burden of deadly heat exacerbated by climate change and existing health inequalities make this a critical location to study.

The burden of adverse pregnancy outcomes (APOs) are mainly felt in low‐ to middle‐income countries, for example an estimated 15 million preterm births (PTB) occur per year, with more than 80% occurring in Asia and sub‐Saharan Africa.^3^ PTB is linked to high rates of both perinatal mortality (the cause of up to 24% of neonatal deaths in sub‐Saharan Africa) and morbidity with long‐term implications.^4^ Triggers for preterm labor are complex and multifactorial, but recent environmental epidemiologic studies demonstrate that maternal exposure to extreme heat increases the risk of PTB.^5^, ^6^ Stillbirths, a neglected tragedy, are again mainly felt in low‐ to middle‐income countries, with increasing rates and have also been linked to extreme heat exposure.^7^

The impact of heat on pregnancy depends on the intensity, duration, and exposure window. First‐trimester exposure leads to increased embryonic death, and cardiac and neurologic anomalies.^8^ In the second and third trimester, maternal exposure to ambient heat has been shown to increase the risk of PTB, stillbirths, and low birth weight in multiple settings.^6^, ^9^, ^10^ Despite strong environmental epidemiologic evidence of the association between heat and APO, there remains limited understanding of the pathophysiologic mechanism associated with these poor outcomes.^11^ One of the proposed hypotheses is that thermoregulatory changes to blood flow prioritize heat loss through cutaneous vasodilatation over other homeostatic mechanisms. For example, in non‐pregnant individuals during exertional heat strain, mesenteric and renal blood flow can be reduced to such an extent that gut permeability or acute kidney injury may occur.^12^ In pregnancy, where blood flow to the uterus and placenta depends on cardiac output, with no autoregulation, there is evidence from animal studies that this occurs,^13^ but human studies are lacking.^11^ However, utero‐placental insufficiency is implicated in the pathophysiologic mechanisms of stillbirth, PTB, and intrauterine growth restriction.^14^ Heat stress could potentially impact on fetal well‐being if the placenta is unable to buffer the effects of the reduction in blood flow leading to transient utero‐placental insufficiency.

Direct measurement of blood flow to the placenta through the uterine arteries can be challenging because it requires highly specialized non‐portable equipment in conjunction with fluid dynamic modeling.^15^ However, the umbilical artery Doppler waveform gives an indication of the fetoplacental circulation function, and so indicates how effectively the fetus is receiving oxygen and nutrients, and removing waste products, and can be used as a surrogate for direct blood flow measurement. The UmbiFlow™ device, a low‐cost portable continuous‐wave Doppler device, was designed and developed in South Africa and has been validated for use to identify placental insufficiency based on the resistance index (RI) of the umbilical artery, with accuracy comparable to commercial units.^16^, ^17^ It has not yet been used to explore dynamic changes in the RI under different physiologic conditions. We hypothesize that heat stress will impact on umbilical artery RI, and those who have APO are more likely to have placental insufficiency under heat stress. Therefore, the following study objectives were defined:

- to determine if the UmbiFlow™ identifies a change in umbilical artery resistance index under heat stress; and
- to determine the practical considerations needed to use UmbiFlow™ in the field.

## 2 MATERIALS AND METHODS

This feasibility study follows the guidelines on reporting non‐randomized pilot and feasibility studies (checklist can be found in the Supplementary material, Table S1).^18^ It was part of a larger prospective cohort study on heat strain in pregnant subsistence farmers and the physiologic impact on their fetuses.^11^ The study was approved by the Gambia Government/MRC Joint ethics committee and the London School of Hygiene and Tropical Medicine Ethics Advisory Board (ref: 16405) in accordance with the Declaration of Helsinki (2013).

Briefly, pregnant women living in West Kiang, The Gambia, participated in an observational cohort study of maternal heat strain and the assessment of the dynamic changes in maternal and fetoplacental blood flow during a day of field work from August 2019 to March 2020, with follow up until December 2020.^19^ Participants were identified through the antenatal clinic or the health and demographic surveillance system in place in West Kiang and were eligible if they were pregnant with a singleton fetus, undertook farming or manual tasks during pregnancy, and did not have pre‐eclampsia or eclampsia at the time of recruitment. Gestational age was determined using last known menstrual period when known, or if unknown, by biparietal diameter on ultrasound scan before 28 weeks of gestation. The feasibility study visits occurred in those with gestational age of 28 weeks or more. Exposure to external environmental conditions (air temperature, relative humidity, solar radiation, wind speed) were measured hourly using the HT200: Heat Stress WBGT Meter, Extech® and the Extech® AN100 thermo‐anemometer (Extech Instruments, Nashua, NH, USA). Measurements were taken within 1 metre of participants to record exact exposure conditions. Two thermal indices were calculated from these measures—the Wet Bulb Globe Temperature (WBGT) and the Universal Thermal Climate Index (UTCI). These are composite measures of thermal stress taking into account heat, humidity, solar radiation, and wind speed.^20^

UmbiFlow™ measures the blood flow velocity in the umbilical cord and calculates the RI as (systolic velocity – diastolic velocity)/systolic velocity. The hand‐held probe attaches to a laptop/tablet, signal processing occurs within the specialized software to give both a waveform and an audible umbilical artery blood flow. Validated reference values by gestational age indicate if the RI is within a normal, intermediate, or high‐risk risk range (RI below 75th centile, between 75th and 95th centiles, and above the 95th centile, respectively, for gestational age). On a single occasion for each participant the RI was measured at baseline in an air‐conditioned environment with the participant supine at rest, and with abdominal lateral tilt, and then at two time points during her working day—determined based on the length of the work shift to correspond with a mid‐point and end‐point of the manual tasks. At each time point, two measurements were taken, assessed for quality (signal quality assessed by expert trained by the South African team), mean values were taken when good/moderate quality and discarded if poor quality. The risk category was recorded at each reading as well as the exact value of the RI. Fetal heart rate was measured concurrently, and average values over 5 min were taken. When fetal heart rate or umbilical artery RI was identified as high risk during the study and did not resolve within 30 min, participants were referred for urgent care. This involved being assessed by the rural antenatal clinic (staffed by a midwife and four doctors), with the option to be referred to a tertiary center should they identify a clinical need. All participants were followed until after delivery and data on birth outcome were collected. APOs were defined as follows: stillbirth—pregnancy longer than 20 weeks where the baby was born dead; PTB—live birth before the completion of 37 weeks of pregnancy; low birth weight—birth weight less than or equal to 2.5 kg; small for gestational age—birth weight 10% below expected weight for gestational age based on Intergrowth‐21 standardized curves.

To determine practical considerations, the study team recorded any issues with the software, the use of UmbiFlow in the field, and the results generated.

All analyses were performed in R version 4.1.0. Descriptive characteristics are presented as mean ± standard deviation or median (interquartile range) by outcome, depending on distribution. The relationship between UTCI, fetal heart rate, and umbilical artery RI were explored using linear and non‐linear models. Non‐linear models were tested across different spline definitions and different knots placed at the median and 90th centile. The lowest Akaike information criterion was used to determine best model fit. Change in fetal heart rate by UTCI was best explained by a linear model. RI *Z* score (which is age adjusted) or change in RI by UTCI was best explained by a non‐linear model with a cubic spline with one knot at the median.

A multilevel linear regression model, with individual as random effect, of the association between umbilical artery RI *Z* score and heat stress was explored both with and without cubic splines, with the best fit determined by Akaike information criterion. The final model is shown as:

$$Z\;{\text{score}}_{\mathrm{ij}}\sim b_{0}+{b_{1}}^{*}\text{heat}\;{\text{stress}}_{\mathrm{ij}}.$$

where *Z* score is umbilical artery RI *Z* score for individual *i* at time *j* and heat stress is UTCI for individual *i* at time *j*.

Multilevel model assumptions were assessed by examining the normality of residuals and performing Levene tests for homogeneity of variance. The simr package was used to run a simulation‐based power analysis on the multilevel model to give estimations of sample size requirements to detect a difference in umbilical artery RI *Z* score under heat stress.

## 3 RESULTS

Full umbilical artery Doppler was completed on 40 participants the field. Of these 40 participants, 17 had APO and 23 did not. Of those with APO, three experienced stillbirths, seven delivered preterm (spontaneously), six were low birth weight, and eight had SGA. Descriptive characteristics of all participants are presented in Table 1, with detailed description of those with stillbirths in Table 2.

**TABLE 1:** Demographic, social, obstetric, and anthropometric characteristics of those with adverse pregnancy outcomes and those without^a^

Characteristics	Adverse pregnancy outcome (*n* = 17)	No adverse outcome (*n* = 23)
Age, year	32.3 ± 7.6	31.6 ± 7.3
Occupation: farmers/other	16/1	16/7
Marital status		
Married	17	21
Single	0	1
Widowed	0	1
Gravida	5 (5.0)	5 (3.5)
Parity	3 (4.0)	4 (3.0)
GA at study visit	31.1 ± 3.1	30.5 ± 2.9
Height, cm	161.2 ± 5.4	162.6 ± 6.3
Weight, kg	62.3 ± 7.7	64.8 ± 12.1
BMI	24.0 ± 2.9	24.5 ± 3.9
Blood pressure	108/72	115/72
Hb, g/dL	11.3 ± 1.1	11.0 ± 1.6
Infection during pregnancy	7/17 (41%)	13/23 (57%)
Pre‐eclampsia/eclampsia	1/17 (6%)	5/18 (28%)
Gestational age at birth, wk	38.4 ± 3.3	40.1 ± 1.7
Birth weight, kg	2.8 ± 0.5	3.4 ± 0.4
Adverse outcomes		
Stillbirths	3/40 (7.5%)	
Preterm births	7/40 (17.5%)	
Low birth weight	6/40 (15%)	
Small for gestational age	8/40 (20%)	

Abbreviations: BMI, body mass index (calculated as weight in kilograms divided by the square of height in meters); GA, gestational age; Hb, hemoglobin.

^a^ Data are presented as mean ± standard deviation, median (interquartile range) or as number (percentage).

**TABLE 2:** Details of participants who had stillbirths

	Case A	Case B	Case C
Maternal age	40–45 years	40–45 years	25–30 years
Gravida/Parity	12/7	10/9	2/1
Previous stillbirth	Yes	No	No
Previous miscarriage	Yes	No	No
Previous neonatal death	No	Yes	No
GA at visit	31 + 6	34 + 5	32 + 1
UA RI – baseline RC	High risk	Low risk	Low risk
UA RI – during work RC	High risk	High risk	Low risk
UA RI – after work RC	High risk	High risk	Low risk
Action	Referred for urgent care	Referred for urgent care	Normal care
GA at delivery	37 + 1	40 + 1	42 + 2
Outcome	Stillbirth	Stillbirth	Likely intrapartum death

Abbreviations: GA, gestational age; RC, risk category (low risk, intermediate risk, high risk based on *z*‐score); UA RI, umbilical artery resistance index.

Environmental conditions and physiologic parameters at baseline, during the work shift, and at the end of the work shift are presented in Table 3 (full exposures can be found in the Supplement Material, Figure S1). All participants were exposed to “extreme heat stress” (based on the UTCI value), which has been shown to increase risk of mortality in other populations and settings.^21^ Average physical energy expenditure for the working shift was equivalent to moderate intensity exercise such as a brisk walk.^22^ There was no significant difference between working environmental conditions or estimated energy expenditure in those who went on to have an APO compared with those who did not.

**TABLE 3:** Environmental conditions, maternal tympanic temperature, fetal heart rate, and umbilical artery resistance index^a^

	Baseline		Mid‐way		End of shift	
	APO	No APO	APO	No APO	APO	No APO
UTCI, °C	23.1 ± 1.3	22.1 ± 2.2	34.2 ± 4.0	33.6 ± 4.2	34.1 ± 3.2	34.5 ± 2.7
WBGT, °C	19.3 ± 1.0	18.6 ± 1.8	27.3 ± 4.1	27.2 ± 4.5	27.3 ± 3.6	27.7 ± 2.7
Air temp., °C	24.0 ± 1.5	22.9 ± 2.0	34.2 ± 3.6	33.9 ± 4.1	34.4 ± 3.2	34.8 ± 3.0
*T*_tym_, °C	36.9 ± 0.2	36.9 ± 0.2	37.2 ± 0.4	37.1 ± 0.3	37.1 ± 0.3	37.3 ± 0.3
PAEE, kcal/kg/h	—	—	—	—	3.1 ± 0.9	3.1 ± 0.7
FHR, beats/min	128.4 ± 8.4	126.4 ± 7.8	142.2 ± 14.4	147.3 ± 7.1	143.7 ± 11.8	144.9 ± 11.5
RI	0.68 ± 0.10	0.65 ± 0.08	0.69 ± 0.10	0.67 ± 0.05	0.66 ± 0.05	0.65 ± 0.07
*Z* score	0.74 ± 1.59	0.43 ± 1.23	0.964 ± 1.58	0.52 ± 0.74	0.51 ± 0.89	0.23 ± 1.13

Abbreviations: APO, adverse pregnancy outcomes; FHR, fetal heart rate; PAEE, physical activity energy expenditure; RI, umbilical artery resistance index; T_tym_, tympanic temperature; UTCI, universal climate thermal index; WBGT, wet bulb globe temperature.

^a^ Data are presented as mean ± standard deviation.

**FIGURE 1:**
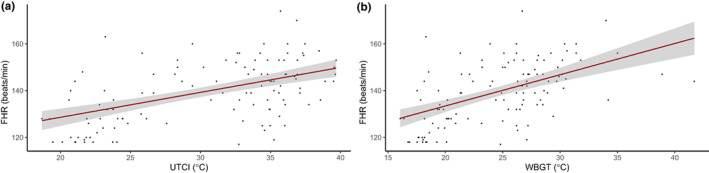
Association between fetal heart rate (FHR) and universal climate thermal index (UTCI) (a), and FHR and wet bulb globe temperature (WBGT) (b). Linear model with 95% confidence interval as shading.

**FIGURE 2:**
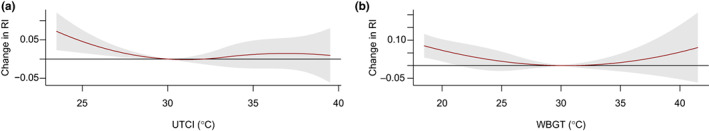
Association between change in umbilical artery resistance index (RI) and universal climate thermal index (UTCI) (a), and wet bulb globe temperature (WBGT) (b). Multilevel model output with 95% confidence interval as shading.

Fetal heart rate demonstrated a linear relationship with heat stress, giving an increase of 10.7 (95% confidence interval [CI] 7.5–13.8) beats/min for each 10°C UTCI increase and 13.4 (95% CI 9.5–17.2) beats/min for each 10°C WBGT increase (Figure 1). However, there was no clear linear or nonlinear relationship between fetal heart rate and maternal tympanic temperature. Change in RI from cool baseline to working conditions reduced with increasing heat stress exposure up to 32°C UTCI/30°C WBGT and then appears to begin to increase with rising heat stress (Figure 2). There was no statistical difference in association between RI and heat stress in those with APO versus without APO (see Supplementary Material, Figure S2). Based on these findings, several simulation‐based power calculations are given in Table 2. Model diagnostics for normality of residuals and homogeneity of variance (using Levene test) did not indicate gross violation of model assumptions.

Practical considerations for use in the field included ensuring a comfortable and private area to scan—which was provided by local vegetation or screens; protection from extreme weather—provided by portable shade/rain protector; and need for accurate gestational age to calculate RI *Z* scores. Several challenges were identified: software compatibility, delay in loading of the program when in the field, and interference with the signal when using windows 7. The programming for the software can only run on windows and is incompatible with Apple devices. It also required a laptop versus a tablet so requiring multiple electronic devices to be carried into the field because all study records were taken directly onto tablets. There was a delay in loading the software at each start up, which required planning for especially considering the need to capture dynamic changes. We also struggled with interference in the signal when using windows 7, which resolved on upgrading to windows 10, but made measurements difficult. All these issues were worked through and we were able to successfully record umbilical artery RI in the field at all the required time points. Furthermore, the manufacturers of UmbiFlow™ are working to resolve many of the points raised.

## 4 DISCUSSION

We show that the UmbiFlow™ device is highly suited to field work, being light and compact, and that the measurement of umbilical artery Doppler in field conditions is possible and shows promising evidence of potentially enhancing the understanding of the fetoplacental circulation response to heat stress. Under heat stress conditions below 32°C UTCI/30°C WBGT there was a reduction in the umbilical artery RI from baseline, which would indicate increased blood velocity within the fetoplacental circulation as we would expect. However, above these temperature thresholds there appears to be a trend towards increasing RI, which would indicate insufficiency in the fetoplacental circulation. The response to heat stress may be different in those individuals that went on to have an APO as shown in the difference in z‐scores presented in Table 3; however, the present study was not powered to determine this with statistical significance.

There are few studies exploring the impact of heat on uterine or placental blood flow. A study from Sweden on sauna use (20 min at 70°C) in late pregnancy found a reactive increase in fetal heart rate, but no change in umbilical artery blood flow.^23^ This study is not immediately translatable to other settings because of both the inactivity and the extreme heat, but could be reassuring in terms of short bursts of unavoidable extreme heat exposure. Other studies have mainly focused on thermoregulation in pregnancy and there are several studies with encouraging evidence that thermoregulation is not compromised.^24^, ^25^ Although there is clear evidence that moderate intensity exercise is of benefit in pregnancy,^26^ these studies are in temperate conditions and so not transferable to our setting. Additionally, in extreme cases (Olympic athletes exercising at more than 90% maximum maternal heart rate) there can be compromised fetal well‐being.^27^ This extreme physiologic strain may be similar to that experienced under extreme heat and warrants further investigation.

The study has several limitations. The sample size was reduced because the COVID‐19 pandemic halted all field work activity from March 2020, limiting the scope of analysis available. Maternal core temperature could not be measured in the field (impractical to use rectal thermometer and lack of evidence on safety for core telemetry pills) and therefore the less accurate and less precise tympanic temperature was measured. Additionally, pregnancy and neonatal outcomes in the general population of The Gambia are worse than the global average, which may impact on the generalizability of the findings globally, although they could be reasonably representative of a rural sub‐Saharan Africa population. We found the number of participants with pre‐eclampsia was higher in those without an APO, but this is most likely due to chance. This study comes at a time when extreme heat exposure is becoming a reality for much of the global population. Despite this, those most commonly experiencing these extreme conditions are often missing from the medical literature. This study is set in a rural African setting, with a population of women that can be difficult to access but are often exposed to extreme environmental conditions. By exploring ways to improve the understanding of pathophysiologic mechanisms in a real‐life setting we highlight the need for future work. The simulation‐based sample size calculations (see Supplementary Material, Table S2) give an estimate of the sample size and conditions needed to progress understanding of this using the UmbiFlow™ device. However, without expanding the work to include several key topic areas, the impacts of this research will have little meaning to this population. Identifying at‐risk women will not be beneficial without clear management options to reduce the risk of these adverse outcomes. Health system strengthening in both facilities and human capacity in dealing with maternal health are urgently needed, especially in the face of the growing climate crisis and resultant impacts on health care. Additionally, identification of a dangerous heat exposure threshold will not have a real‐world impact until evidence‐based, effective, realistic, pragmatic, and sustainable interventions for cooling both individuals and their environment are identified and enacted.

## AUTHOR CONTRIBUTIONS

AB contributed to conceptualization, methodology, formal analysis, and writing the original draft. VV contributed to the methodology, validation, and editing. BS contributed to software, data curation, and editing. NM contributed to formal analysis and editing. AVC, AH, NM, JH, and AP contributed to conceptualization, methodology, supervision, and editing.

## FUNDING INFORMATION

This project was funded by the Wellcome Trust through the Wellcome Trust Global Health PhD Fellowship awarded to AB (216336/Z/19/Z). The funders had no role in study design, data collection, analysis, manuscript writing, or decision to submit.

## CONFLICT OF INTEREST

The authors have declared that there are no conflicts of interest.

## Supporting information


Data S1

## Data Availability

Anonymized data will be made available on reasonable request from the corresponding author.
