# Supplementary material for: A feasibility study of the use of UmbiFlow™ to assess the impact of heat stress on fetoplacental blood flow in field studies
Source: Int J Gynaecol Obstet. 2022 Oct 10;160(2):430–6. doi: 10.1002/ijgo.14480 (PMC10092781; doi:10.1002/ijgo.14480)
Supplement: Supplementary file 1 — Data S1 [file IJGO-160-430-s001.docx]

**SUPPLEMENT**

**A feasibility study of the use of UmbiFlow™ to assess the impact of heat stress on fetoplacental blood flow in field studies.**

Ana Bonell, Valerie Vannevel, Bakary Sonko, Nuredin Mohammed, Ana M. Vicedo-Cabrera, Andy Haines, Neil S Maxwell, Jane Hirst, Andrew M Prentice

Table of contents:

| Table 1: STROBE checklist | 2 |
| --- | --- |
| Table 2: Simulation based power calculations | 6 |
| Figure 1a: Time series of heat stress (WBGT) exposure | 7 |
| Figure 1b: Time series of heat stress (UTCI) exposure | 7 |
| Figure 2a: Change in umbilical artery RI under heat stress (WBGT) in those with and without APO | 8 |
| Figure 2b: Change in umbilical artery RI under heat stress (UTCI) in those with and without APO | 9 |

STROBE Statement—checklist of items that should be included in reports of observational studies

|  | Item No. | Recommendation | Page  No. | Relevant text from manuscript |
| --- | --- | --- | --- | --- |
| **Title and abstract** | 1 | (*a*) Indicate the study’s design with a commonly used term in the title or the abstract | 1 | Feasibility study in title |
|  |  | (*b*) Provide in the abstract an informative and balanced summary of what was done and what was found | 2 | See abstract |
| Introduction | | | |  |
| Background/rationale | 2 | Explain the scientific background and rationale for the investigation being reported | 3 | With the ongoing climate crisis, global extreme heat exposure is progressively increasing…… |
| Objectives | 3 | State specific objectives, including any prespecified hypotheses | 5 | determine if UmbiFlow™ identifies a change in umbilical artery resistance index under heat stress; and  determine the practical considerations needed to use UmbiFlow™ in the field |
| Methods | | | |  |
| Study design | 4 | Present key elements of study design early in the paper | 6 | Briefly, pregnant women living in West Kiang, The Gambia, participated in an observational cohort study |
| Setting | 5 | Describe the setting, locations, and relevant dates, including periods of recruitment, exposure, follow-up, and data collection | 6 | …from August 2019 to March 2020, with follow-up until December 2020. |
| Participants | 6 | (*a*) *Cohort study*—Give the eligibility criteria, and the sources and methods of selection of participants. Describe methods of follow-up  *Case-control study*—Give the eligibility criteria, and the sources and methods of case ascertainment and control selection. Give the rationale for the choice of cases and controls  *Cross-sectional study*—Give the eligibility criteria, and the sources and methods of selection of participants | 6 | Participants were identified through the antenatal clinic or the health and demographic surveillance system in place in West Kiang and were eligible if they were pregnant with a singleton, undertook farming or manual tasks during pregnancy and did not suffer with pre-eclampsia or eclampsia at the time of recruitment. |
|  |  | (*b*) *Cohort study*—For matched studies, give matching criteria and number of exposed and unexposed  *Case-control study*—For matched studies, give matching criteria and the number of controls per case |  |  |
| Variables | 7 | Clearly define all outcomes, exposures, predictors, potential confounders, and effect modifiers. Give diagnostic criteria, if applicable | 6-7 | External environmental conditions (air temperature, relative humidity, solar radiation, wind speed) were measured hourly using the HT200: Heat Stress WBGT Meter, Extech® ….. |
| Data sources/ measurement | 8* | For each variable of interest, give sources of data and details of methods of assessment (measurement). Describe comparability of assessment methods if there is more than one group | 6-7 | UmbiFlow™ measures the blood flow velocity in the umbilical cord and calculates the RI = (systolic velocity – diastolic velocity)/systolic velocity |
| Bias | 9 | Describe any efforts to address potential sources of bias | 7 | Duplicate measurements of RI taken to reduce bias and measurement error |
| Study size | 10 | Explain how the study size was arrived at | NA | Study to determine needed sample size. Sample size of cohort study based on expected rates of heat stress and heat strain – see protocol paper |

| Quantitative variables | 11 | Explain how quantitative variables were handled in the analyses. If applicable, describe which groupings were chosen and why | 7-8 | Exposure variables continuous and not grouped. Outcome variable defined |
| --- | --- | --- | --- | --- |
| Statistical methods | 12 | (*a*) Describe all statistical methods, including those used to control for confounding | 8 | Multilevel model used |
|  |  | (*b*) Describe any methods used to examine subgroups and interactions |  |  |
|  |  | (*c*) Explain how missing data were addressed |  |  |
|  |  | (*d*) *Cohort study*—If applicable, explain how loss to follow-up was addressed  *Case-control study*—If applicable, explain how matching of cases and controls was addressed  *Cross-sectional study*—If applicable, describe analytical methods taking account of sampling strategy | NA |  |
|  |  | (*e*) Describe any sensitivity analyses |  |  |
| Results | | | | |
| Participants | 13* | (a) Report numbers of individuals at each stage of study—eg numbers potentially eligible, examined for eligibility, confirmed eligible, included in the study, completing follow-up, and analysed | 8-9 | Table 1 |
|  |  | (b) Give reasons for non-participation at each stage |  |  |
|  |  | © Consider use of a flow diagram |  |  |
| Descriptive data | 14* | (a) Give characteristics of study participants (eg demographic, clinical, social) and information on exposures and potential confounders | Table 1 | Table 1 |
|  |  | (b) Indicate number of participants with missing data for each variable of interest |  |  |
|  |  | © *Cohort study*—Summarise follow-up time (eg, average and total amount) | 7 | All participants followed up after delivery…. |
| Outcome data | 15* | *Cohort study*—Report numbers of outcome events or summary measures over time | Table 1 | Table 1 |
|  |  | *Case-control study—*Report numbers in each exposure category, or summary measures of exposure |  |  |
|  |  | *Cross-sectional study—*Report numbers of outcome events or summary measures |  |  |
| Main results | 16 | (*a*) Give unadjusted estimates and, if applicable, confounder-adjusted estimates and their precision (eg, 95% confidence interval). Make clear which confounders were adjusted for and why they were included | 9 |  |
|  |  | (*b*) Report category boundaries when continuous variables were categorized | NA |  |
|  |  | (*c*) If relevant, consider translating estimates of relative risk into absolute risk for a meaningful time period |  |  |

| Other analyses | 17 | Report other analyses done—eg analyses of subgroups and interactions, and sensitivity analyses | Supplement | Fig 2 |
| --- | --- | --- | --- | --- |
| Discussion | | | | |
| Key results | 18 | Summarise key results with reference to study objectives | 11 | We show that the UmbiFlow™ device is highly suited to field work, being light and compact and that |
| Limitations | 19 | Discuss limitations of the study, taking into account sources of potential bias or imprecision. Discuss both direction and magnitude of any potential bias | 12 | Several limitations….. |
| Interpretation | 20 | Give a cautious overall interpretation of results considering objectives, limitations, multiplicity of analyses, results from similar studies, and other relevant evidence | 13 | Additionally, identification of a dangerous heat exposure threshold….. |
| Generalisability | 21 | Discuss the generalisability (external validity) of the study results | 12 | Additionally, pregnancy and neonatal outcomes in the general population of The Gambia are worse than the global average which may impact on generalisability of the findings globally but could be reasonably representative of a rural SSA population |
| Other information | |  | | |
| Funding | 22 | Give the source of funding and the role of the funders for the present study and, if applicable, for the original study on which the present article is based | 14 |  |

*Give information separately for cases and controls in case-control studies and, if applicable, for exposed and unexposed groups in cohort and cross-sectional studies.

**Note:** An Explanation and Elaboration article discusses each checklist item and gives methodological background and published examples of transparent reporting. The STROBE checklist is best used in conjunction with this article (freely available on the Web sites of PLoS Medicine at http://www.plosmedicine.org/, Annals of Internal Medicine at http://www.annals.org/, and Epidemiology at http://www.epidem.com/). Information on the STROBE Initiative is available at [www.strobe-statement.org](http://www.strobe-statement.org).

**Table 2:** Power calculation predictor for determining association between umbilical artery resistance index z-scores under heat stress.

| Outcome | Highest UTCI exposure (°C) | Power to detect effect size (%) | 95% CI | Sample size |
| --- | --- | --- | --- | --- |
| Association between RI and heat stress undifferentiated | 34.79 | 83% | 80.53;85.28 | 500 |
| Association between RI and heat stress in those with APO | 34.92 | 91.1 | 89.2;92.8 | 997 |

**
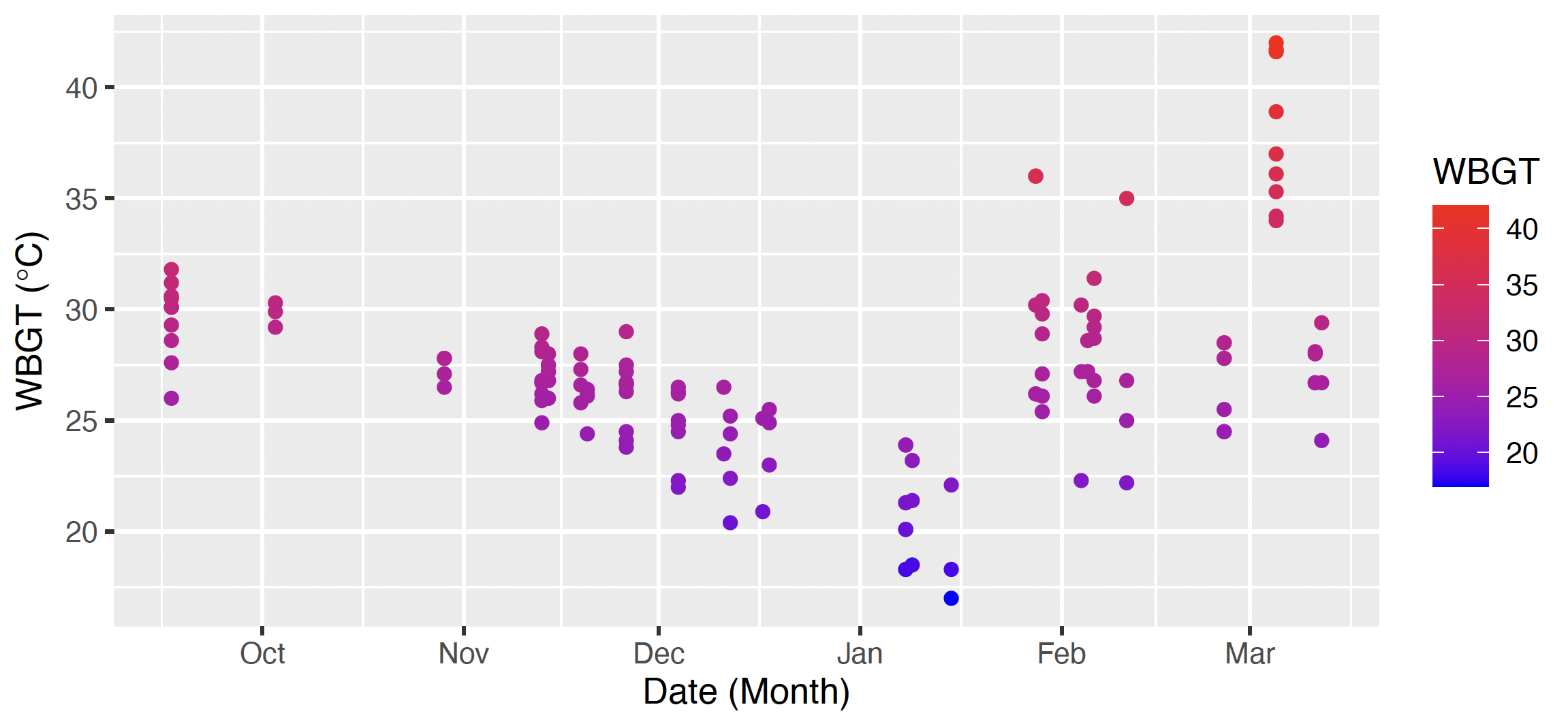
Figure 1a:** Time series of heat stress (Wet Bulb Globe Temperature - WBGT) exposure. Each point corresponds to a directly observed measurement during the field study.

**Figure 1b:** Time series of heat stress (Universal Thermal Climate Index - UTCI) exposure. Each point corresponds to a directly observed measurement during the field study.

**Figure 2a:** Change in umbilical artery RI under heat stress (WBGT) in those with and without APO. Black = no APO; red = APO. Shading = 95% CI.

**Figure 2b:** Change in umbilical artery RI under heat stress (UTCI) in those with and without APO. Black = no APO; red = APO. Shading = 95% CI.
